# Biomechanics of Copenhagen Adduction Exercise and Eccentric Hip Adductor Exercises on a Novel Isokinetic Dynamometer With Within‐Session Repeatability

**DOI:** 10.1111/sms.70365

**Published:** 2026-09-02

**Authors:** J. Marušič, O. Cvjetičanin, M. Supej, J. Babič, N. Šarabon

**Affiliations:** ^1^ Faculty of Health Sciences University of Primorska Izola Slovenia; ^2^ Faculty of Sport University of Ljubljana Ljubljana Slovenia; ^3^ Department for Automatics, Biocybernetics and Robotics, Laboratory for Neuromechanics and Biorobotics Jožef Stefan Institute Ljubljana Slovenia; ^4^ Ludwig Boltzmann Institute for Rehabilitation Research Vienna Austria

**Keywords:** eccentric training, groin injury, injury prevention, joint torque, strength

## Abstract

This study biomechanically evaluated the eccentric phase of short and long lever Copenhagen adduction exercise (CAE) and compared them with unilateral and bilateral eccentric hip adductor exercises performed on a novel eccentric hip adductor dynamometer (EHAD). Fifteen participants completed two CAE variations and five EHAD exercises. Three‐dimensional kinematics and force data were collected to compute peak hip joint torque, range of motion (ROM), and angle at peak torque as the primary dependent variables. Peak and mean external force and body‐mass‐normalized torque were reported. Effects of exercise type and lever length on primary dependent variables, as well as relative and absolute within‐session repeatability, were evaluated. The CAE produced significantly lower peak eccentric torques than all EHAD variations, regardless of lever length or unilateral/bilateral performance. Long lever CAE elicited greater peak and average torques than the short lever CAE, although ROM was significantly lower in the long lever condition across exercises. EHAD exercises generated significantly greater peak torques during bilateral performance, demonstrating the presence of bilateral facilitation (27.9% ± 16.5%). Angle at peak torque was similar across all exercises (31°–39°). With the exception of angle at peak torque during the CAE, all measurements showed good to excellent relative and moderate to good absolute within‐session repeatability. Increasing lever length during CAE effectively raises hip adductor loading, while all EHAD variations enable even greater loading and exhibit pronounced bilateral facilitation. Future intervention studies should determine whether these biomechanical advantages translate into clinically meaningful improvements in adductor‐related groin injury prevention and rehabilitation outcomes.

## 1 Introduction

Groin injuries present a significant problem in sport, accounting for 18% of all time loss injuries and affecting about one in five players per season in men's professional football, with a substantial recurrence burden of 17.7%–21% [1, 2, 3]. The most common groin injury is the adductor strain, which accounts for approximately two‐thirds of all groin injuries [1, 4, 5, 6], while previous groin injury, higher level of play, lower levels of sport‐specific training, and reduced hip adductor strength are associated with an increased risk of groin injury [7]. These injuries can lead to substantial time loss and decreased performance, making effective preventive measures essential. For instance, data from professional football players reported a median time loss of 10 days per groin injury (IQR: 5–22 days) [1].

A training intervention using the Copenhagen adduction exercise (CAE) has been shown to reduce the risk of football players reporting groin pain during the competitive season by 41% and the average weekly prevalence during the competitive season by 7.8% compared to the control group [8]. The CAE is performed in the side‐lying position, with the athlete supporting their weight on the elbow and placing the upper leg on an elevated surface or, in a partner‐assisted variation, supported by a teammate (as implemented in the intervention study by Harøy et al.) [8]. Exercise intensity can be modified by changing the lever length: placing the knee on the support for a short lever variation or the ankle for a long lever variation. The exercise strengthens the hip adductors through hip abduction (eccentric phase) and adduction (concentric phase) while maintaining a neutral hip position (0°) in the sagittal plane. Previous research has shown that the CAE can sufficiently load the hip adductors to elicit high electromyographic activity of the adductor longus (108% ± 5% of maximal voluntary isometric contraction) [9] and significantly increase eccentric strength (by 36% after an 8‐week intervention in underage amateur football players) [10], which is likely the main reason for its preventive efficacy. Adding to this evidence, recent 8‐week intervention studies examining frequency and volume effects showed that, in high‐level youth rink hockey players, twice weekly CAE produced larger isometric strength improvements than once weekly (+24% vs. low volume; +34% vs. controls) [11]; and in female football players, both low and high volume CAE‐based programmes increased isometric hip adduction strength (+6.2%–12.2%) with no between‐group differences [12]. However, when compared with an alternative hip adductor strengthening approach (adductor squeeze isometric exercise), an 8‐week CAE programme did not produce superior gains in isometric or eccentric strength, highlighting that comparable strength improvements may also be achievable with other targeted adductor exercises in sub‐elite, recreational football players [13]. Additionally, adding the CAE to an exercise‐based 8‐week rehabilitation programme performed twice weekly resulted in a greater improvement in eccentric hip adduction strength than rehabilitation alone (≈46% vs. ≈24%), alongside greater reductions in pain and disability [14].

Despite these intervention benefits, detailed biomechanical characterization of the CAE remains limited. While peak external force during the eccentric lowering phase has recently been quantified using an externally fixed load cell setup [15], the corresponding hip joint torques, the hip angle at which peak torque occurs, the exercise range of motion (ROM), and the influence of lever length on these parameters remain largely unknown. Additionally, certain practical and biomechanical features of the CAE should be considered when interpreting the extent of its potential preventive effect. Specifically, it does not allow (a) exclusively eccentric contractions, (b) eccentric contractions under a (supra)maximal load, or (c) eccentric contractions across the full range of hip abduction (i.e., full stretch of the hip adductors). Hamstring injury prevention programmes incorporating the eccentric Nordic hamstring exercise are associated with an approximately 50% reduction in hamstring injury risk [16, 17], although subgroup analyses in meta‐analyses have not consistently shown that including the Nordic hamstring exercise further increases the preventive effects of exercise programmes [18]. Broader reviews also identify eccentric strengthening as an effective measure for reducing future hamstring injury risk, particularly when compliance with the programme is high [19]. Given the effectiveness of eccentric training for hamstring injury prevention and the identical injury mechanism of these muscles to that of the hip adductors (explosive muscle activation during rapid muscle lengthening) [20], we hypothesize that high load eccentric training through the full ROM may represent a relevant preventive approach and warrants comparison with the concentric–eccentric loading of the CAE in future intervention studies. However, the detailed mechanisms of eccentric training for the hip adductors remain largely unexplored. To address this and enable controlled evaluation of eccentric hip adductor loading we developed a new eccentric hip adductor dynamometer (EHAD), described in detail in the Methods section (see Figure 2), which measures the torque–angle relationship during bilateral or unilateral eccentric contractions of the hip adductors.

The primary aim of this study was to biomechanically evaluate and compare the eccentric phase of the CAE (long and short lever) to various eccentric hip adductor exercises on the EHAD (long and short lever, unilateral and bilateral versions). The primary dependent variables used to assess the main effects and interaction of exercise were peak joint torque, hip angle at peak torque, and ROM. In addition, average and peak external forces, and body‐mass‐normalized torques were reported to facilitate comparison with previous studies and to support interpretation in clinical settings. A secondary aim was to evaluate the relative and absolute within‐session repeatability of the measured parameters.

## 2 Materials and Methods

### 2.1 Participants

Sixteen recreationally active participants volunteered for this study: 10 males (age: 28.6 ± 4.2 years, height: 1.84 ± 0.05 m, mass: 81.6 ± 8.8 kg, BMI: 24.2 ± 2.2 kg/m^2^) and 6 females (age: 24.8 ± 4.4 years, height: 1.66 ± 0.04 m, mass: 60.6 ± 5.4 kg, BMI: 22.0 ± 1.3 kg/m^2^). The statistical power analysis tool G*Power 3.1. software [21] was used to calculate the minimum required sample size under the following assumptions: a medium effect size (*f* = 0.25), a statistical power of 80%, and a significance level of α = 0.05 for a repeated measures ANOVA. The analysis showed that a minimum sample size of 15 participants was required. Inclusion criteria required the absence of any musculoskeletal injuries or pain syndromes within the previous year and the ability to perform two sets of five repetitions of the long lever CAE. Prior to the start of the measurements, participants were thoroughly informed about the details of the protocol and were required to sign an informed consent form. The protocol was conducted in accordance with the latest revision of the Declaration of Helsinki. The experimental procedures were reviewed and approved by the Commission of the University of Primorska for Ethics in Human Subjects Research (KER UP) (reference number: 4264‐19‐6/23).

### 2.2 Study Design and Procedures

This cross‐sectional study required participants to perform the CAE and eccentric exercises for the hip adductors on the EHAD (described in more detail below) after a standardized warm‐up. All participants were familiarized with both exercise types beforehand. The warm‐up consisted of light aerobic activity (6 min of alternating stepping on a 25 cm high box), dynamic stretching and strengthening exercises (hip circles, squats, side lunges, hip bridges, sit‐ups and hip extensions), and eccentric contractions on the dynamometer with subjective gradation of intensity at 25%, 50%, 75% and 90% of maximal voluntary effort. Prior to the warm‐up set, maximal ROM for the EHAD was determined individually by passively guiding each participant into hip abduction until a slight yet painless stretch was perceived.

**FIGURE 1:**
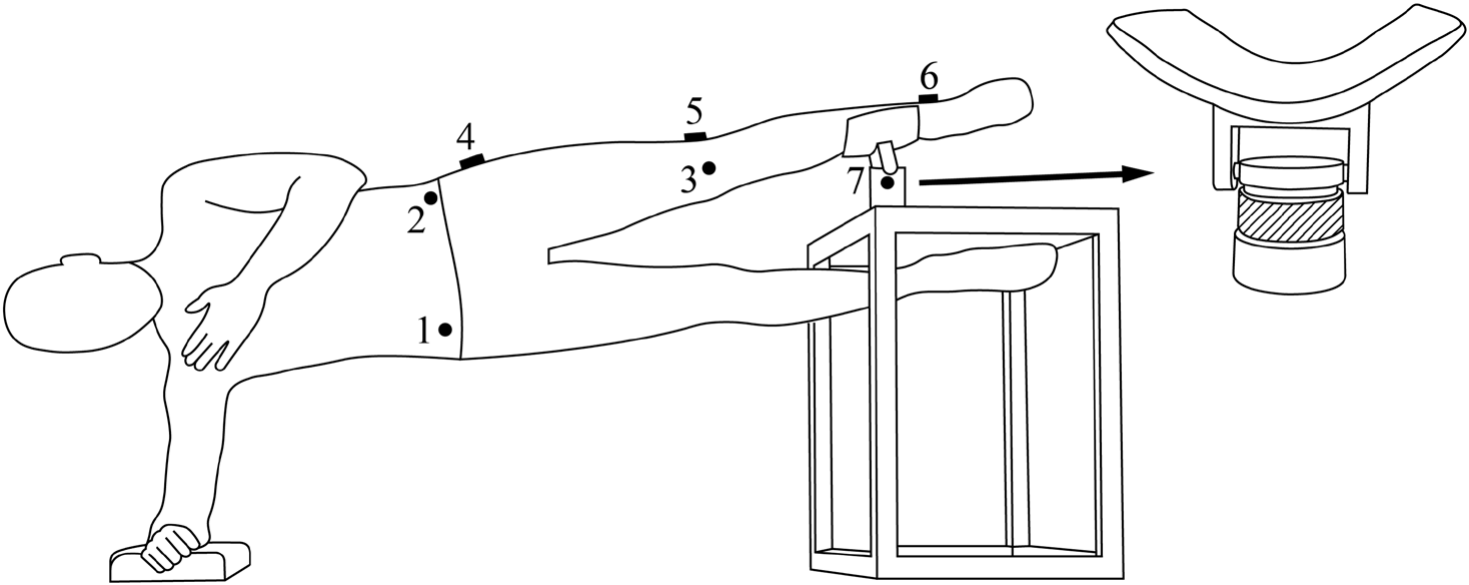
Overview of the experimental setup for the Copenhagen adduction exercise on a padded platform attached to an axle located directly above the built‐in force sensor (the sensor is shown as the shaded part on the right side of the image). Placement of kinematic markers: 1, 2 (SIAS); 3 (patella); 4 (greater trochanter); 5 (lateral epicondyle); 6 (lateral malleolus); 7 (force sensor).

Kinematic markers were placed bilaterally on the anterior superior iliac spines and unilaterally on the greater trochanter, patella, lateral epicondyle, lateral malleolus of the measured leg, and on the force sensor, as shown in Figure 1. Participants performed one set of five repetitions of both the long lever (ankle on the elevated surface) and short lever (knee on the elevated surface) CAE with the dominant leg in a randomized order. The height of the elevated surface was individually set to match the participant's greater trochanter height in standing. The reason for this is practical, as the CAE is normally performed on the (sports) field without additional equipment and in pairs of approximately equal height. In this case, when the individual holds the teammate's loaded leg in an upright, standing position, the height of that leg is approximately at the level of the greater trochanter. The measured leg rested either just above the medial malleolus (long lever) or just above the medial epicondyle (short lever) on a padded platform mounted on an axle positioned just above the built‐in 6‐axis force sensor (Optoforce HEX‐H, International Federation of Robotics, Frankfurt, Germany) (Figure 1). In addition to force, three‐dimensional kinematics (3D Optotrak Certus System, NDI Inc., Ontario, Canada) were also recorded synchronously.

**FIGURE 2:**
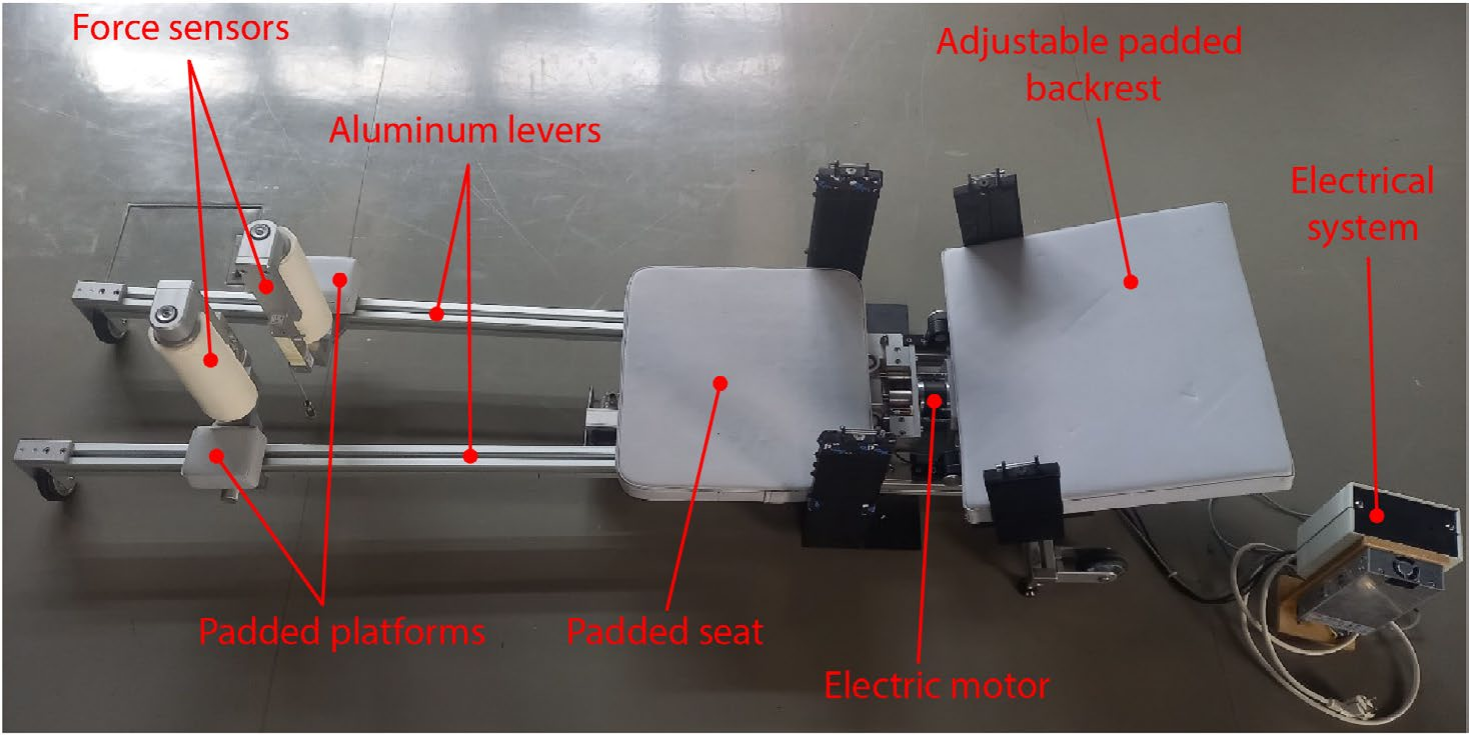
Isokinetic dynamometer for eccentric loading of hip adductors.

The eccentric exercises were performed on the EHAD (shown in Figure 2), which was designed to enable bilateral or unilateral isokinetic eccentric contractions of the hip adductors. This device has also been used previously to examine eccentric hip adduction torque across different hip flexion angles [22]. The exercises were performed at an angular velocity of 13°/s (fixed due to motor‐related design constraints). This velocity allows most participants to reach the end ROM in hip abduction within approximately 3 s of eccentric loading. Participants lay supine with their feet (long lever) or knees (short lever) placed on padded platforms connected to aluminium levers. These levers were moved by a 24 V DC electric motor (TT Motor Industrial Co. Ltd., Shenzhen, China), forcing the participants into hip abduction. The padded platforms were equipped with two force sensors (PW2DC3, HBK, Darmstadt, Germany) and could be adjusted to match the lever length, either just above the ankle or just above the knee. The electric motor was powered and controlled by a custom‐designed electrical system, allowing straightforward operation of the device. The hip abduction angle was measured through the change in displacement of the aluminium levers in relation to the starting position via a linear transducer (SX80, WayCon, Munich, Germany).

**FIGURE 3:**
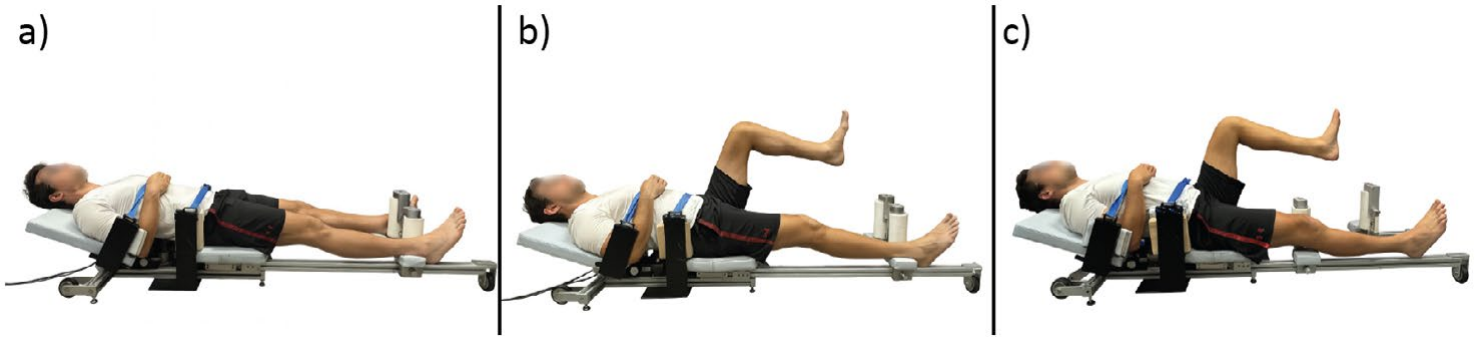
Position of the participant on the isokinetic dynamometer for eccentric loading of hip adductors during (a) bilateral variation with long lever, (b) unilateral variation with long lever, and (c) unilateral variation with short lever of eccentric exercise for hip adductors.

Five eccentric variations were performed in randomized order, each consisting of five repetitions with 5 min of rest: bilateral variations with long lever (the padded platforms were placed just above the medial malleolus) and short lever (the padded platforms were placed just above the medial epicondyle), unilateral variations with long and short lever on the dominant leg, and unilateral variation with long lever on the non‐dominant leg. Before each trial, participant positioning was visually checked and adjusted using the greater trochanter as a practical bony landmark to approximate alignment between the hip joint axis and the device axis. Participants were instructed to exert maximal resistance throughout the full range of each repetition as the dynamometer moved their legs into hip abduction. In the unilateral version, the participants held the unmeasured leg in the air with a hip and knee flexion of 90°. Fixation straps were placed over the pelvis and chest to stabilize the upper body (shown in Figure 3). During the measurements, the participants received strong verbal encouragement. After a 30‐min rest, within‐session repeatability was assessed for the long lever CAE, bilateral long lever variation on EHAD, and unilateral long lever variation on EHAD. Short lever variations were not repeated to avoid excessive fatigue.

### 2.3 Signal Processing

During the CAE, kinematic data on the positions of markers from the Optotrak system were recorded at a frequency of 100 Hz, while the forces acting on the leg from the dynamometer were measured at a frequency of 450 Hz. The force data were hardware‐synchronized with the kinematic data and resampled to 100 Hz using third‐order spline interpolation. Signals were smoothed using a zero‐lag fourth‐order low‐pass Butterworth filter with a cut‐off frequency of 3 Hz. The cut‐off frequency was chosen based on the fact that the CAE was performed as a slow, non‐impact motion, with each repetition lasting approximately 3–4 s. The selected cut‐off frequency was roughly one order of magnitude higher than the fundamental frequency of the movement, and visual analysis verified that the signals under examination preserved the integrity and morphological characteristics. The hip joint center was determined from marker positions according to Bell et al. [23]. The hip joint angle in the frontal plane was defined as the angle between the vector formed by the left and right SIAS and the vector formed by the hip joint center and the patella. Although kinematic and kinetic data were acquired in three dimensions, the inverse dynamics analysis for the CAE was reduced to the frontal plane. Hip adduction and abduction torque were quantified as the net internal torque about the anterior–posterior axis passing through the estimated anatomical hip joint center. Segment inertial parameters of the measured leg were estimated from the anthropometric tables of Winter [24], including segment mass, center of mass location, and moment of inertia. Specifically, the net internal hip torque was computed utilizing a Newton–Euler inverse dynamics framework applied to a free‐body model of the measured limb segments. In this approach, the sum of forces acting on each segment was set equal to the segment mass multiplied by linear acceleration, and the sum of moments was set equal to the segment's moment of inertia multiplied by angular acceleration. External torques, gravitational influences, and inertial effects of the limb segments were incorporated into the calculations, although inertial contributions were anticipated to be minimal. The biomechanical model accounted for variations in segment configuration between the long lever and short lever CAE setups. It was assumed in the calculation that the point of external force application on the leg was at the extension of the force vector acting through the dynamometer's pivot point. Specifically, the dynamometer recorded the external force as a three‐dimensional force vector, which was subsequently projected onto the frontal plane for analysis. A kinematic marker affixed to the force sensor assembly of the dynamometer was utilized to determine the position of the dynamometer pivot point within the Optotrak coordinate system. Given that the padded support was mounted on a freely rotating hinge, the line of action of the external force was assumed to intersect this pivot point. The external hip torque was computed as the vector product of the vector originating from the estimated hip joint center to the point of application of the external force and the projected external force vector. Each repetition of the CAE was defined as the eccentric part of the movement performed at constant velocity. The primary dependent variables were the peak hip torque in the frontal plane, ROM, and the hip angle at peak torque during the CAE. In addition, average torque, average and peak external force, and body‐mass‐normalized force and torque were reported.

The force and angle signals from the EHAD were acquired at a sampling frequency of 1000 Hz and processed using dedicated ARS software (S2P, Science to Practice Ltd., Ljubljana, Slovenia). The signals were low‐pass filtered using a fourth‐order Butterworth filter with a cut‐off frequency of 20 Hz. The highest mean force over a 0.1 s window was taken and multiplied by the lever distance to obtain joint torque. The lever distance was defined as the distance from the device rotation axis to the center of the padded force platform. The moment arm was treated as perpendicular to the force vector throughout the ROM, and no angle‐dependent correction was applied. The primary dependent variables were the peak hip torque in the frontal plane, ROM, and the hip angle at peak torque. In addition, peak external force, and body‐mass‐normalized torque were reported. Furthermore, the bilateral index (BI) was calculated according to Howard and Enoka [25] as $\mathrm{BI}=\left(100\cdot \frac{T_{B}}{T_{\mathrm{UR}}+T_{\mathrm{UL}}}\right)-100$, where *T*_*B*_ represents the sum of torques produced by each leg during the bilateral task, and *T*_*UR*_ and *T*_*UL*_ represent the torques produced by the right and left leg, respectively, during unilateral tasks. Positive values indicate bilateral facilitation, whereas negative values indicate a bilateral deficit.

### 2.4 Statistical Analysis

The analyses were carried out using SPSS statistical software (version 26.0, IBM: Armonk, NY, USA). The best repetition (the one with the highest peak torque) of the dominant leg was used for the statistical analysis. Descriptive statistics were calculated and reported as mean ± standard deviation. The normality of the data distributions for all variables was verified with the Shapiro–Wilk test. A two‐way ANOVA for repeated measurements (3 × 2) was conducted to assess the main effects and interaction of exercise (bilateral variation on the EHAD, unilateral variation on the EHAD, the CAE) and leg lever (long lever, short lever) on the primary dependent variables (peak joint torque, hip angle at peak torque, and ROM). Mauchly's test of sphericity was used to test the sphericity assumption, and if violated, the Greenhouse–Geisser correction was applied. Partial eta squared (η^2^) effect sizes were calculated and interpreted as small (0.01–0.06), medium (0.06–0.13) and large (> 0.14). When significant main effects were found, Bonferroni‐adjusted paired *t*‐tests were used for post hoc comparisons. Cohen's d effect sizes were calculated and interpreted as trivial (< 0.2), small (0.2–0.5), medium (0.5–0.8), large (0.8–1.2), very large (1.2–2.0), and huge (> 2.0) [26]. Relative within‐session repeatability was assessed using intraclass correlation coefficient based on absolute agreement, two‐way random effects and a single measure (peak value) model (ICC_2.1_) and interpreted as follows: values below 0.5 as poor repeatability, values between 0.5 and 0.75 as moderate repeatability, values between 0.75 and 0.9 as good repeatability, and values above 0.9 as excellent repeatability. Absolute within‐session repeatability was assessed by calculating the typical error of measurement and coefficient of variation (CV), which was interpreted as poor (CV > 10%), moderate (CV = 5%–10%) and good (CV < 5%) [27]. The level of statistical significance was set at α = 0.05.

## 3 Results

All but one participant successfully performed all planned variations of the CAE and eccentric exercises on the EHAD. One participant did not complete the measurements due to the onset of pain in the lumbar spine during the CAE with long lever. Therefore, data from 15 participants were used for the following results, and descriptive statistics are presented in Table 1.

**TABLE 1:** Descriptive statistics (mean ± standard deviation).

	CAE LL	CAE SL	EHAD BI LL	EHAD BI SL	EHAD UNI LL	EHAD UNI SL
Peak force (*N*)	203.4 ± 35.7	265.6 ± 47.4	267.2 ± 52.8	552.1 ± 106.7	212.9 ± 42.5	468.1 ± 97.7
Peak force nor (*N*/kg)	2.8 ± 0.5	3.7 ± 0.6	3.7 ± 0.5	7.6 ± 1.4	2.9 ± 0.4	6.5 ± 1.2
Peak torque (Nm)	121.9 ± 29.5	81.5 ± 17.6	206.6 ± 47.6	204.2 ± 41.5	165.0 ± 40.5	173.1 ± 37.7
Peak torque nor (Nm/kg)	1.7 ± 0.2	1.1 ± 0.1	2.8 ± 0.4	2.8 ± 0.4	2.3 ± 0.4	2.4 ± 0.4
Angle at peak torque (°)	30.9 ± 9.6	34.3 ± 11.9	36.7 ± 9.0	39.0 ± 9.7	39.1 ± 11.0	38.7 ± 8.3
ROM (°)	36.5 ± 6.9	41.2 ± 8.6	44.2 ± 9.6	45.6 ± 8.7	44.2 ± 10.0	45.6 ± 8.7

Abbreviations: BI, bilateral variation; CAE, Copenhagen adduction exercise; EHAD, eccentric hip adductor dynamometer; force nor, force normalized with body mass; LL, long lever; ROM, range of motion; SL, short lever; torque nor, torque normalized with body mass; UNI, unilateral variation.

**FIGURE 4:**
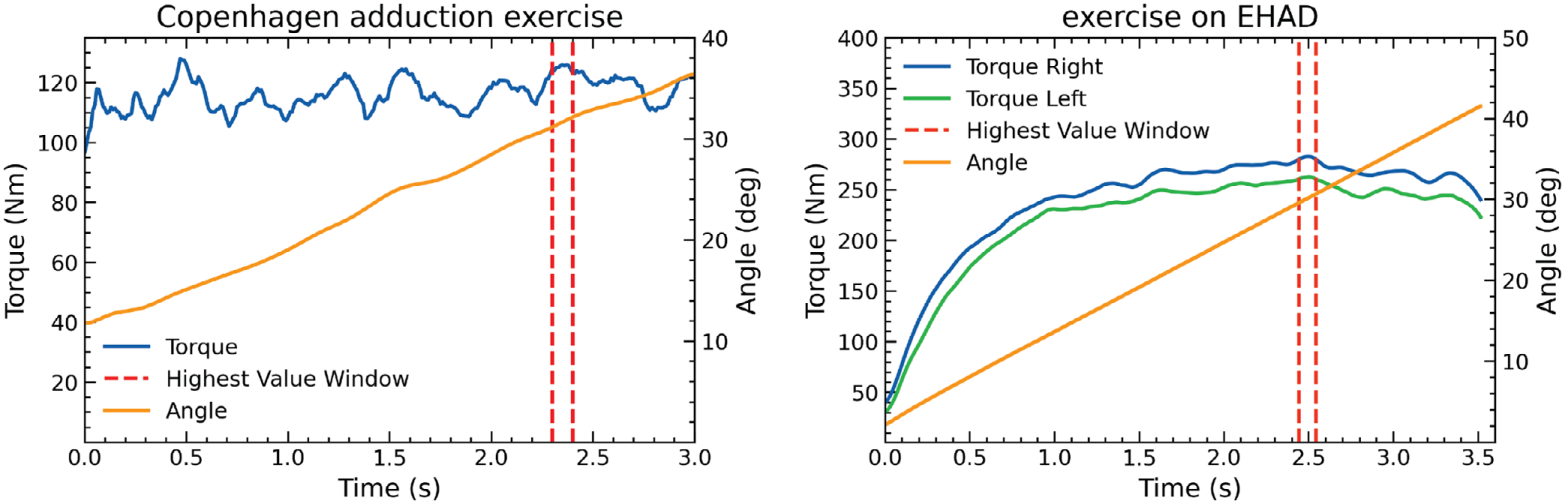
A representation of a typical torque and hip angle values during the eccentric phase of the long lever Copenhagen adduction exercise (left side) and a long lever bilateral exercise on the EHAD (eccentric hip adductor dynamometer) (right side). The blue and green lines represent the torque of the right and left legs, respectively. The yellow line represents the hip angle, and two dotted red lines indicate the position of the peak torque (highest mean value at a window length of 0.1 s).

The average angular velocity was 9.0° ± 3.0°/s for the CAE at long lever and 9.2° ± 3.0°/s for the short lever, compared with the fixed eccentric speed of the EHAD (13°/s). A representative example of torque and joint angle for one repetition of the CAE and bilateral eccentric exercise on EHAD is shown in Figure 4.

### 3.1 Within‐Session Repeatability

Relative and absolute within‐session repeatability between the best repetitions (based on highest peak torque value) from each of the two sets is shown in Table 2. Overall, within‐session repeatability was good to excellent for force, torque and ROM across all conditions (ICC ≥ 0.78; CV ≤ 10.36%), whereas the angle at peak torque showed poor repeatability during the CAE (ICC = 0.12; CV = 23.9%). Systematic bias between sets was mostly non‐significant, with effect sizes ranging from trivial to medium (Cohen's *d* = 0.00–0.67). An exception was average force during the CAE, which showed a significant difference with a large effect size (*p* = 0.001, *d* = 1.03). Significant differences were also observed for peak force (*p* = 0.047, *d* = 0.56) and ROM (*p* = 0.021, *d* = 0.67) during the CAE.

**TABLE 2:** Within‐session repeatability.

		Mean ± SD Set 1	Mean ± SD Set 2	TEM	ICC_2.1_	CV	Sys. bias	
							Sig.	Cohen's *d*
Copenhagen adduction exercise	Peak force (*N*)	203.4 ± 35.7	197.9 ± 35.2	6.89	0.97	3.44	0.047	0.56
	Avg force (*N*)	196.5 ± 36.5	189.3 ± 35.3	4.92	0.98	2.55	0.001	1.03
	Peak torque (Nm)	121.9 ± 29.5	117.8 ± 26.9	5.98	0.96	4.99	0.083	0.48
	Avg torque (Nm)	110.2 ± 27.0	106.6 ± 25.7	4.95	0.97	4.57	0.067	0.51
	Angle at peak torque (°)	30.9 ± 9.6	35.6 ± 7.0	7.94	0.12	23.87	0.127	0.42
	ROM (°)	36.5 ± 6.9	40.2 ± 8.9	3.97	0.78	10.36	0.021	0.67
EHAD‐bilateral	Peak force (*N*)	267.2 ± 52.8	259.2 ± 54.8	19.0	0.89	7.21	0.270	0.30
	Peak torque (Nm)	206.6 ± 47.6	200.4 ± 47.7	15.26	0.91	7.50	0.282	0.29
	Angle at peak torque (°)	36.7 ± 9	36.8 ± 10.5	4.81	0.78	13.07	0.988	0.00
	ROM (°)	44.2 ± 9.6	44 ± 9.8	0.85	0.99	1.92	0.547	0.16
EHAD‐unilateral	Peak force (*N*)	212.9 ± 42.5	216.8 ± 49.3	19.1	0.85	8.91	0.584	0.14
	Peak torque (Nm)	165 ± 40.5	168 ± 44.7	15.12	0.89	9.08	0.600	0.14
	Angle at peak torque (°)	39.1 ± 11	39.8 ± 9.8	3.61	0.90	9.16	0.640	0.12
	ROM (°)	44.2 ± 10	44.6 ± 9.6	0.63	1.00	1.43	0.064	0.52

Abbreviations: CAE, Copenhagen adduction exercise; CV, coefficient of variation; EHAD, eccentric hip adductor dynamometer; ICC, intraclass correlation coefficient; ROM, range of motion; SD, standard deviation; TEM, typical error of measurement.

### 3.2 Effects of Exercise (Dynamometer‐Bilateral, Dynamometer‐Unilateral, CAE), Leg Lever (Long, Short), and Their Interaction

A 3 × 2 repeated measures ANOVA was conducted to assess the effects of exercise (EHAD‐bilateral, EHAD‐unilateral, CAE), leg lever (long, short), and their interaction on peak torque, angle at peak torque, and ROM. Results are shown in Table 3. Overall, peak torque showed significant effects of exercise, lever, and their interaction; ROM showed a significant effect of lever only, and angle at peak torque showed no significant effects.

**TABLE 3:** Results of a 3 × 2 repeated measures ANOVA.

Parameter	Effect	*F*	*p*	*η* ^2^
Peak torque (Nm)	Lever	8.23	0.012*	0.37
	Exercise	121.56	< 0.001*	0.90
	Exercise × lever	19.23	< 0.001*	0.58
Angle at peak torque (°)	Lever	1.54	0.235	0.10
	Exercise	2.60	0.125	0.16
	Exercise × lever	0.83	0.446	0.06
ROM (°)	Lever	10.05	0.007*	0.42
	Exercise	3.86	0.069	0.22
	Exercise × lever	2.86	0.110	0.17

*Note:* * denotes statistical significance.

Abbreviations: *η* ^2^, partial eta squared effect size; *F*, statistic value for the repeated measures ANOVA; ROM, range of motion.

### 3.3 Peak Torque

As shown in Table 3, the analysis revealed significant main effects for exercise and for lever. Furthermore, a significant interaction effect between exercise and leg lever on peak joint torque was observed, suggesting that the differences between the levers were dependent on the exercise.

**FIGURE 5:**
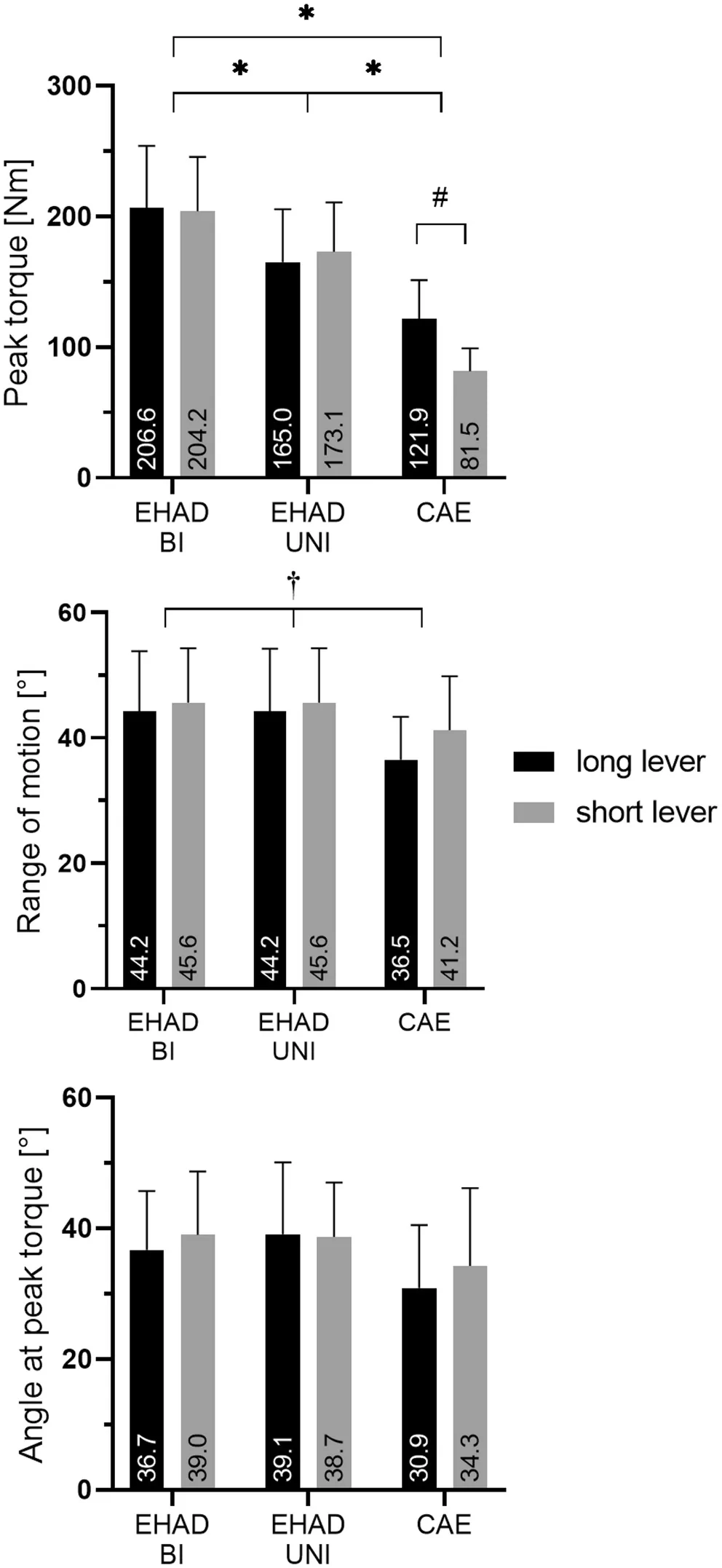
Peak torque, range of motion, and angle at peak torque across six different exercises. CAE, Copenhagen adduction exercise; EHAD BI, bilateral exercises on the dynamometer for eccentric loading of hip adductors; EHAD UNI, unilateral exercises on the dynamometer for eccentric loading of the hip adductors; # denotes a statistically significant difference between two CAE variations; * denotes a statistically significant difference between three long lever exercises and between three short lever exercises; † denotes a significant main effect of leg lever on ROM.

Post hoc pairwise comparisons revealed a significant difference between short and long lever CAE; specifically, long lever CAE produced significantly higher joint torque compared to short lever CAE (+49.6%, *p* < 0.001, *d* = 2.52) (Figure 5). In contrast, there was no significant difference between the two bilateral variations (*p* = 0.697) or between the two unilateral variations on EHAD (*p* = 0.327).

A further analysis was performed to compare the three exercises for each lever separately with one‐way repeated measures ANOVA. There were significant differences between exercises with long lever (F(2, 28) = 55.47, *p* < 0.001, *η* ^2^ = 0.80). Post hoc pairwise comparisons revealed that bilateral variation on EHAD produced significantly higher joint torque compared to unilateral variation on EHAD (+25.2%, *p* < 0.001, *d* = 1.37) and compared to CAE (+69.5%, *p* < 0.001, *d* = 2.49). Additionally, unilateral variation on EHAD produced significantly higher joint torque compared to CAE (+35.4%, *p* < 0.001, *d* = 1.48).

Similarly, significant differences between exercises with short lever were found (F(2, 28) = 136.41, *p* < 0.001, *η* ^2^ = 0.91). Post hoc pairwise comparisons revealed that bilateral variation on EHAD produced significantly higher joint torque compared to unilateral variation on EHAD (+18.0%, *p* = 0.001, *d* = 1.18) and compared to CAE (+150.6%, *p* < 0.001, *d* = 3.76). Additionally, unilateral variation on EHAD produced significantly higher joint torque compared to CAE (+112.4%, *p* < 0.001, *d* = 3.02) (Figure 5).

### 3.4 Range of Motion

There was no significant main effect of exercise on ROM and no significant interaction effect between exercise and leg lever. However, there was a significant main effect of leg lever on ROM, with greater ROM in the short lever condition (44.1° ± 6.4°) than in the long lever condition (41.6° ± 6.6°) across all exercises (Figure 5).

### 3.5 Angle at Peak Torque

There was no significant main effect for leg lever, for exercise and no significant interaction effect on angle at peak torque (Figure 5). Therefore, the additional within‐lever one‐way repeated measures ANOVA was considered exploratory. It showed significant differences between exercises with long lever F(1.44, 20.12) = 4.06, *p* = 0.045, *η* ^2^ = 0.23. However, post hoc pairwise comparisons showed no significant differences in angle at peak torque between the long lever variations (*p* = 0.097–0.630).

### 3.6 Bilateral Index

The summed peak torques of the left and right leg during the unilateral long lever variations were 329.4 ± 81.0 Nm, whereas the summed peak torques obtained during the bilateral variation reached 416.8 ± 95.2 Nm. The resulting bilateral index averaged 27.9% ± 16.5%, indicating bilateral facilitation (the 95% confidence interval ranged from 19.6% to 36.2%).

## 4 Discussion

To our knowledge, this is the first study to biomechanically evaluate the CAE and compare it to isokinetic eccentric exercises for the hip adductors. In summary, the results demonstrated that: (a) peak torque produced during the eccentric phase of CAE is significantly lower compared to eccentric exercises on the EHAD, irrespective of lever length and bilateral or unilateral performance; (b) peak torque produced during bilateral exercises on the EHAD is significantly greater compared to unilateral exercises on the EHAD, irrespective of lever length; (c) peak torque produced during the long lever CAE is significantly greater compared to the short lever CAE; (d) ROM is significantly greater in the short lever than in the long lever condition, while the angle at peak torque remains relatively consistent across all exercises; (e) except for the angle at peak torque during the CAE, all measurements show good to excellent relative and moderate to good absolute within‐session repeatability.

Until recently, biomechanical evaluation of the CAE had been limited to elicited EMG activity [9]. However, two recent studies have also quantified external forces during CAE variations: Hickey et al. [15] reported peak external force during the eccentric lowering phase of a long lever CAE (325–328 N), and O'Connor et al. [28] reported peak force during a 5‐s isometric long lever CAE, including a weighted progression (≈313 N with body mass only, increasing with added external load). In our setup, peak external force during the long lever CAE was lower (203 N), which may partly reflect differences in the measurement approach and exercise execution between studies. However, the most likely explanation is the difference in participant characteristics, particularly body mass and sex distribution in our sample (including females with mean body mass ≈60 kg), compared with the substantially heavier male cohort in O'Connor et al. (male rugby players with mean body mass ≈96 kg) [28].

The results of our study provide additional new insights into other biomechanical features, including the torque profile across the eccentric phase, the hip angle at which peak torque occurs, and ROM, as well as the effect of lever length. Specifically, the long lever CAE generated average torque of 110.2 ± 27.0 Nm (1.5 ± 0.2 Nm/kg) throughout the eccentric phase with a peak torque of 121.9 ± 29.5 Nm (1.7 ± 0.2 Nm/kg). As expected, both values were significantly lower in the short lever CAE, which produced an average torque of 72.5 ± 17.6 Nm (1.0 ± 0.1 Nm/kg) and a peak torque of 81.5 ± 17.6 Nm (1.1 ± 0.1 Nm/kg). The results confirm that changing the lever length of the CAE is a convenient way to adjust the loading intensity for the hip adductors. However, the increased load comes at the expense of a significantly reduced ROM, as participants reached the terminal (bottom) position sooner due to floor contact rather than flexibility limitations. Reduced ROM may represent a factor to consider for further optimization of the exercise's preventive effect, as it limits loading at longer muscle–tendon lengths, where most adductor‐related groin injuries occur [20].

Another limitation of the CAE is that its loading depends on body mass, which may be insufficient for most (elite) athletes. In such cases, CAE training programmes are usually intensified by increasing the number of repetitions, which typically means sets of 15 repetitions in the last week of the prescribed 8‐week training protocol [8, 10, 12, 13]. However, this combination of load intensity and high repetition volume may not optimally target maximal eccentric hip adductor strength, which has been identified as one of the main risk factors for groin injuries [7, 29]. Recent findings also show that adding weights to the isometric CAE enables athletes to generate significantly greater external force (up to 48% greater than the bodyweight CAE) [28]. Future CAE interventions incorporating systematic progression with added external load would therefore be valuable to evaluate whether a higher intensity stimulus improves strength adaptations and preventive effects.

From a biomechanical perspective, exercises performed on the EHAD may provide a different eccentric training stimulus, as they enable high‐intensity eccentric contractions throughout the full ROM and, as demonstrated in the present study, induce significantly higher loads on the hip adductors (165–206 Nm; 2.3–2.8 Nm/kg) in comparison to the CAE. In this regard, and given their isolated eccentric loading, EHAD exercises share some similarities with the Nordic hamstring exercise, which has been shown to be effective in hamstring injury prevention [16, 17]. Given that hip adductor strains share the same underlying injury mechanism as hamstring strains (explosive muscle activation during rapid muscle lengthening in a stretched position, e.g., change of direction, kicking, reaching and jumping [20]), such high‐intensity eccentric loading could also be relevant for adductor‐related groin injury prevention, although this remains to be confirmed. Specifically, eccentric training (compared to concentric‐eccentric) may result in (a) a higher mechanical load on the muscle–tendon complex, leading to greater maximal eccentric strength gains [30, 31], and a better tolerance to high eccentric loads during specific high‐risk sport situations, (b) a shift in the angle at peak torque toward a greater muscle–tendon length [32], (c) serial sarcomerogenesis [33], and (d) an improvement in flexibility [34, 35]. However, it remains uncertain whether these adaptations occur, and to what extent, following long‐term eccentric‐only hip adductor training, and how such adaptations compare with those achieved through CAE‐based interventions. Additionally, the eccentric exercises in the present study were performed at slow velocities and therefore do not replicate the rapid muscle lengthening observed during sport‐specific situations in which injuries typically occur. Thus, the potential preventive relevance of these and other similar eccentric exercises, such as the Nordic hamstring exercise, may be more strongly linked to the capacity to produce high eccentric torque (throughout the full ROM), rather than to movement velocity.

High‐intensity eccentric loading may also be relevant in rehabilitation following acute adductor injuries, where deficits in eccentric strength can persist into the later stages of return to sport preparation. In a prospective study of male athletes completing a standardized, criteria‐based rehabilitation programme, eccentric hip adduction strength improved progressively and explained a meaningful proportion of progression toward return to sport milestones [36]. Given the substantial recurrence burden after hip adductor injuries in elite football (17.7% to 21% recurrences [1, 2, 3]), incorporating appropriately progressed high‐intensity eccentric loading into rehabilitation may help support a safer return to sport and potentially reduce reinjury risk. Further (intervention) studies are required to test these hypotheses regarding hip adductor‐related groin injury prevention and rehabilitation training.

On the EHAD, participants achieved peak torque near the end of the ROM (37°–39°), which is consistent with findings from a study using isokinetic unilateral concentric contractions of the hip adductors [37]. Surprisingly, peak torque during the CAE also occurred near the end of the ROM; the joint angles at peak torque were similar across all six exercise variants (31°–34° of hip abduction during the CAE vs. 37°–39° during exercises performed on EHAD). Theoretically, peak torque during the CAE should occur closer to the neutral hip position (i.e., 0°) as this is when the effective torque lever is longest. During the eccentric phase, as the hip is lowered and the angle of abduction increases, the lever is progressively shorter, leading to reduced torque. However, the results of our study showed the opposite, with the peak torque occurring closer to the final ROM. One possible explanation is the muscle force‐length relationship. At larger hip abduction angles, the adductors operate at longer muscle–tendon lengths, which has been shown to increase force generating capacity [22, 37] and thereby influence the angle at peak torque. Additionally, it is likely that the lower the participant brought the hip during the eccentric phase, the more the participant had to stabilize themself with their upper limb and push toward the sensor to maintain the required position. Importantly, the 6‐axis force sensor captured not only vertical forces but also horizontal shear components, which increased as stabilization demands rose near terminal hip abduction. This occurred because the leg support was unstable, since it was mounted on an axle just above the sensor and rotated freely without friction as it adjusted to the changing angle at the hip joint. This required participants to generate additional stabilizing forces, which contributed to the measured torque and shifted the peak toward the terminal position. Furthermore, although the movement velocity during the CAE was relatively low (9.0°–9.2°/s), the dynamics associated with approaching the terminal ROM may also have contributed to the observed torque profile. As the participant neared the bottom position, the movement had to be decelerated and subsequently reaccelerated in the opposite direction. This braking and reacceleration demand may contribute to slightly elevated torque toward the end of the eccentric phase. However, this demand is unlikely to have substantially influenced the present results, as only the constant velocity portion of the eccentric phase of the CAE was included in the analysis. In addition, the difference between the average and peak torque during the long lever CAE was in fact small (110.2 ± 27.0 vs. 121.9 ± 29.5 Nm, respectively), and the torque was relatively consistent across the whole ROM, unlike the torque profile observed during exercises on EHAD (Figure 4). It can be inferred that the peak torque during the standard CAE variation (where the active limb is on a stable and immobile support instead of the moving brace) would behave somewhat differently and more closely resemble the theoretical torque–angle profile, where peak torque angle would occur closer to 0°.

In addition to producing higher torques through a greater ROM, performing the exercise on the EHAD is also a more time‐efficient training method, as it enables bilateral performance in contrast to the unilateral CAE. Nevertheless, the CAE still has the advantage in terms of overall practicality, as it can be performed with a partner without requiring any additional equipment or devices. Furthermore, its impact on the abdominal muscles should not be overlooked, as maintaining a side plank position throughout the set may enhance the endurance of the lateral abdominal muscles and contribute to improved trunk stabilization (assuming the athlete's body mass provides a sufficient stimulus). Interestingly, unilateral EHAD exercises may also involve meaningful abdominal muscles co‐contraction, because it is difficult to fully stabilize the pelvis in isolation using external straps alone. To achieve complete pelvis stabilization for maximal unilateral eccentric contraction, additional trunk fixation and abdominal muscle activation are also required. However, because electromyographic activity was not recorded, the magnitude of abdominal muscle activity on the EHAD relative to the CAE remains unknown and warrants further investigation. Additionally, full‐range eccentric exercise with supramaximal loading may exacerbate both the onset and severity of delayed onset muscle soreness, requiring greater attention to training progression. This consideration is particularly important when evaluating the feasibility of incorporating such demanding eccentric loading into practical athletic environments and within the broader context of an athlete's overall training process.

When comparing results with other studies that evaluated hip adduction peak torques using unilateral isokinetic testing, the peak torques achieved on the EHAD were substantially higher, particularly in the bilateral condition. For example, healthy young adults achieved 115.3 ± 43.4 Nm during the eccentric phase in the standing position at 60°/s [38], young elite football players (17.5 ± 0.3 years old) achieved 129.10 ± 24.18 Nm during the eccentric contraction in the side‐lying position at 30°/s [39] while participants in our study reached 165 ± 40 Nm during the unilateral and 206.6 ± 47.6 Nm during the bilateral variation. Possible reasons for the discrepancies are differences in the measurement procedure (measurement speed, position, ROM, sample of participants, etc.), but probably the biggest reason is the unilateral‐bilateral nature of the measurement. It appears that when participants perform bilateral eccentric contractions, they are able to produce greater peak torque compared to unilateral contractions. This is called the bilateral facilitation, a phenomenon where maximal bilateral force production is greater than the sum of unilateral forces, and is generally less common compared to the bilateral deficit, which is the exact opposite [40]. In the case of eccentric contractions of the hip adductors in the supine position on the EHAD, this is probably due to the increased requirement for upper body stabilization during unilateral contractions compared to bilateral ones. In the absence of any upper body stabilization, such unilateral contraction is not possible, as the participant would also move their trunk in the opposite direction. To avoid this, two fixation straps were used across the pelvis and torso to ensure the best possible upper body fixation. At the same time, the participants were encouraged to use their arms to additionally stabilize themselves as much as possible during the contractions. Similarly, the phenomenon of bilateral facilitation during hip adductor isometric contractions has been reported in other studies [41, 42, 43], with values ranging widely (10%–100%). In our study, we were the first to demonstrate that this phenomenon also occurs during eccentric contractions, with an observed index of 27.9% ± 16.5%. The results indicate that the EHAD can uniquely provide greater eccentric loading compared with conventional isokinetic dynamometers, especially in the bilateral condition. This has important implications not only for training but also for strength assessment and risk‐factor monitoring. One commonly recommended approach for preventive screening is the measurement of “eccentric” hip adductor strength using hand‐held dynamometry in a break test, typically performed in or near the neutral frontal plane position (0°) [44, 45]. However, such testing does not capture the muscle's eccentric capacity in the joint positions where adductor injuries most frequently occur; namely at substantially lengthened muscle–tendon lengths close to the end of the available ROM. In contrast, eccentric strength assessed in the final ~15° of hip abduction may provide biomechanically more relevant information and potentially represent a more accurate risk factor for adductor‐related groin injury.

Biomechanical dependent variables generally showed good to excellent relative within‐session repeatability (ICC = 0.78–1.00) and moderate to good absolute within‐session repeatability (CV = 1.4%–10.4%) across all exercise variations. Notable exceptions were the angle at peak torque during the bilateral performance on EHAD (CV = 13.1%) and, in particular, during the CAE, where within‐session repeatability was poor (CV = 23.9%, ICC = 0.12). This reduced repeatability likely reflects the greater instability during the CAE, where the freely rotating support brace introduced additional uncontrolled movement that directly affects the measured joint angle. Additionally, the close similarity between peak and average torque suggests that torque production remains relatively constant throughout the eccentric phase, indicating that the CAE provides a more uniform load profile rather than a distinct torque peak tied to a specific joint position, which may further contribute to poor within‐session repeatability of the angle at peak torque.

Some limitations of this study should be acknowledged. This study involved a heterogeneous sample of physically active adults, which limits the direct generalization of the findings to elite or highly trained athletes. However, as all participants were able to correctly perform at least two sets of five repetitions of the CAE and given that the loading during the CAE is primarily constrained by the body mass, it is unlikely that the basic torque–angle characteristics would differ substantially in well‐trained athletes, even though their absolute strength levels may be higher. Additionally, the biomechanical analysis of the CAE was conducted using a custom setup in which the active limb was supported by a freely rotating axle with an integrated force sensor. While this design enabled continuous torque monitoring, it introduced instability not present in the standard CAE performed on a stable surface. Consequently, the torque–angle relationship observed in this study may not fully correspond to that of the traditional exercise, particularly near terminal hip abduction. Nevertheless, average eccentric torque is unlikely to differ meaningfully from that of the standard (stable) CAE, as the load is primarily determined by body mass, while only the torque–angle profile may differ to a greater extent. Moreover, torque calculation during the CAE is also limited by approximating the hip joint center using pelvic marker regression, a method subject to an anticipated error of 1.9 ± 1.2 cm [23]. This localization uncertainty alters the effective lever arm lengths and subsequently the absolute hip torques, exerting a proportionally larger impact on the short lever than the long lever CAE configuration. Furthermore, because joint angles were obtained using different measurement systems for CAE (kinematic data) and EHAD (linear transducer), between exercise comparisons of joint angles should be interpreted with some caution. In addition, the average CAE angular velocity (about 9°/s) was slightly lower than the fixed EHAD velocity (13°/s), so small differences related to the force–velocity relationship between tasks cannot be excluded. However, given that both are low absolute velocities and represent slow, controlled eccentric contractions, any such influence is likely minimal. Finally, the EHAD used in the present study was a custom‐built research prototype and is currently not commercially available. Therefore, its use is limited to settings with appropriate engineering resources or research collaboration, whereas the CAE remains the most accessible option for practitioners.

## 5 Perspective

This study provides the first biomechanical comparison between the CAE and isokinetic eccentric exercises for the hip adductors, including different lever lengths as well as unilateral and bilateral performance modes. The CAE generated moderate torques during the eccentric phase that increased when performed with a long lever, although this condition was accompanied by a significantly reduced ROM across exercises. In contrast, the EHAD produced significantly greater torques under both unilateral and bilateral conditions, with the present findings also demonstrating the occurrence of bilateral facilitation. Given the established role of high‐intensity eccentric training in eliciting functional and architectural adaptations, the ability of the EHAD to apply such loading across the full ROM may provide a distinct training stimulus that could be relevant for adductor‐related groin injury prevention and rehabilitation. While the CAE remains a practical and accessible option, its loading capacity is inherently limited by body mass and progression is often achieved primarily through increased volume. Future intervention studies are needed to determine whether these biomechanical differences translate into clinically meaningful improvements in prevention and rehabilitation outcomes across different athletic populations.

## Funding

This study was supported by the University of Primorska through internal research programme KINSPO (Kinesiology for movement performance and musculoskeletal injury prevention in sport; grant number: P5‐0443). Additionally, Jan Babič received support from the European Union's Horizon Europe through the project SWAG (contract number 101120408), and by the Slovenian Research Agency (research core funding number P2‐0076).

## Conflicts of Interest

The authors declare no conflicts of interest.

## Data Availability

The data that support the findings of this study are openly available in Dataset: Biomechanics of Copenhagen adduction exercise at https://zenodo.org/records/17863720.
